# Reconstructing RFT through the Lens of the Interbehavioral Field: What is a Relational Frame Anyway?

**DOI:** 10.1007/s40614-025-00485-x

**Published:** 2025-11-24

**Authors:** Dermot Barnes-Holmes, Linda J. Hayes, Colin Harte, Mitch Fryling

**Affiliations:** 1 Ulster University, Coleraine, Northern Ireland; 2 University of Nevada, Reno, NV USA; 3 Universidade Federal de São Carlos, São Carlos, Brazil; 4 Instituto Par—Ciências e Tecnologia de Comportamento, São Paulo, Brazil; 5 Instituto Nacional de Ciência E Tecnologia Sobre Comportamento, Cognição E Ensino, São Paulo, Brazil; 6 California State University, Los Angeles, CA USA

**Keywords:** RFT, Interbehaviorism, Kantor, Field-based analyses

## Abstract

The recent resurgence of Kantorian interbehavioral psychology in the context of relational frame theory (RFT) has prompted a reevaluation of RFT’s core concepts through an interbehavioral lens. Although RFT acknowledges its Kantorian roots, recent works have called for a more serious consideration of interbehaviorism in the context of developing the theory towards a more complete analysis of the complexity of human language and cognition. In particular, the current article aims to explore the alignment between the RFT concept of the relational frame and the interbehavioral interpretation of psychological happenings. To this end, the relational frame is dissected to clarify (mentalistic) misconceptions of RFT, and is then compared with interbehavioral constructs such as stimulus and response functions, substitute stimulation, and interbehavioral history. The integration of these perspectives suggests that RFT may benefit from a field-based approach to experimental and applied research. We argue that by applying the interbehavioral concept of stimulus substitution for stimuli that differ arbitrarily in multiple ways (i.e., multiple stimulus relations), the door may be opened for the entire RFT research program to yield (at least potentially) to interbehavioral field-based analyses.

In recent years, a number of authors have highlighted that the roots of relational frame theory (RFT; Hayes et al., 2001) lie partly in Kantorian interbehavioral psychology (e.g., Barnes-Holmes et al., 2020; Harte & Barnes-Holmes, 2024). For example, the prologue to the 2001 seminal RFT text contained an explicit acknowledgement to a Kantorian influence and indeed the book itself was dedicated to both Skinner and Kantor for forging “... the way toward a naturalistic account of human language and cognition” (Hayes et al., 2001). Furthermore, it is worth noting that the first published explication of the core RFT idea was co-authored by a Kantorian/interbehavioral scholar, L. J. Hayes (Hayes & Hayes, 1989). In recent publications, the focus on Kantorian interbehavioral thinking in RFT has reemerged in what some authors have described as “updating” the theory (Barnes-Holmes & Harte, 2022; Barnes-Holmes, et al., 2020; Harte & Barnes-Holmes, 2024). Providing a detailed review of the work involved in that “updating” is not the purpose of the current article. Nevertheless, the recent refocusing on interbehavioral thinking in RFT, which has been driven by the need to deal with relational complexity, has led us to revisit some of the core concepts in the theory to determine how closely (or not) they align with interbehavioral field-based theorizing in general. In this vein, we anticipate the need to write multiple articles on this topic in order to gain a clearer view of most, if not all, of the key concepts in RFT through the lens of interbehavioral thinking. In beginning this journey, we will focus on the core concept of the theory itself: the relational frame.

The current article will begin, therefore, by reviewing in detail the definition of the relational frame as articulated in the seminal volume. We will then consider a relatively recent RFT-based research program that commenced by focusing on the relative strength or probability of relational framing itself. As will become clear, this research program involved focusing on the interaction among multiple relational frames, and thus greater relational complexity than the analysis of individual relational frames. In grappling with such complexity, the potential utility of an interbehavioral analysis became apparent (Harte & Barnes-Holmes, 2024), which we will consider in the next section. Having done so, we will then explore whether the more basic RFT concept of the relational frame itself similarly yields to an interbehavioral analysis. Although it may seem odd to begin with interbehavioral analyses of relational complexity (i.e., Harte & Barnes-Holmes, 2024), before then returning to the more basic construct of the relational frame, doing so most accurately reflects the intellectual journey that led us to the current point.

## Definition of the Relational Frame

Needless to say, the first task before us is to provide a description of the concept of the relational frame. In doing so, we have drawn on the first book-length treatment of RFT (Hayes et al., 2001). In that text, the following definition is provided:

The term *relational frame* was coined to designate particular kinds of relational responding (Hayes & Hayes, 1989). A relational frame is a specific class of arbitrarily applicable relational responding that shows the contextually controlled qualities of mutual entailment, combinatorial mutual entailment, and transformation of stimulus functions; is due to a history of relational responding relevant to the contextual cues involved; and is not solely based on direct [e.g., explicitly reinforced] non-relational training with regard to the particular stimuli of interest, nor solely to nonarbitrary [formal or physical] characteristics of either the stimuli or the relation between them. (p. 33; text in square brackets here and below is added to original quotation)

In addition, the authors pointed out that “relational frames are a unit of responding and a specific class of functional behavior, but it is wrong to think of this [unit] in mechanical and physicalistic terms” (p. 34), because this would undermine the functional-analytic nature of the unit itself. Furthermore, the authors added the qualification that “... while the generic [or general] concept of a relational frame is foundational to RFT, the concept of any particular relational frame [such as coordination or difference] is not” (p. 40). We will now elaborate on this definition in the service of our later examination of the concept through the lens of interbehavioral field-based theorizing.

According to the quotation above, relational frames are defined by the three properties of mutual entailment, combinatorial mutual entailment, and transformation of stimulus functions. Mutual entailment refers to the emergence of unreinforced or derived relational responding between two stimuli (i.e., reinforcing an A–B relational response in a matching-to-sample procedure, produces derived, unreinforced B–A relational responding). Combinatorial entailment refers to the emergence of unreinforced or derived relational responding involving two overlapping mutually entailed relational responses (i.e., reinforcing A–B relational responding and B–C relational responding in a matching-to-sample procedure, produces derived unreinforced A-C and C-A relational responding). Transformation of stimulus functions refers to changes in the functions of the stimuli that may occur in accordance with mutual and combinatorial entailment. Imagine, for example, that stimulus A has some specific function. When A becomes related to C via combinatorial entailment, the functions of C will be supplemented in some manner, given appropriate contextual cues (see below), based on the specific function of A. According to RFT, the entailment and transformation properties of relational framing are under two classes of contextual control, Crel and Cfunc. The former refers to the stimuli that determine the class of entailment involved in any instance of relational responding. For example, the Crels “is the same as,” “is different from,” “is opposite to,” provide the relational context for particular patterns of relational framing (i.e., coordination, opposition, difference). The latter cue (Cfunc) refers to stimuli that select the relevant stimulus property or properties that will be changed in accordance with the entailed relations. For example, if you are told that A tastes very sweet and B is the opposite to A (in terms of taste), then you may assume that B will taste sour.

In defining a relational frame in terms of two types of contextual control, the explanation for this control is provided by a history of appropriate multiple exemplar training. For example, it is through a young child’s interaction with the verbal community that the appropriate contextual functions for stimuli such as “tastes” (Cfunc) and “same as” (Crel), are established in the child’s behavioral repertoire. In other words, the relevant contextual control requires a history of exposure to appropriate operant contingencies within the verbal community before derived (unreinforced) relational responding is possible. In this sense, the concept of the relational frame is inherently historical and functional, and is thus conceptualized as a generalized operant class. As such, relational framing, as a type of operant class, is not considered a new behavioral *principle*; however, RFT has invoked the concept of a new behavioral *process* because “of the logically necessary implications of such operants” (Hayes & Barnes-Holmes, 2004, p. 215). That is, RFT posits that the operant behavior of relational framing alters the functions of other behavioral processes. To put it another way, relational framing is operant behavior that impacts the process of operant learning itself.

Overall, therefore, the generic concept of the relational frame appears undeniably functional^1^ because it incorporates any individual pattern of relational framing—for example, same, opposite, different—but is not defined by any one of these specific frames. This, of course, is consistent with the concept of an operant, which may include, for example, lever pressing or key pecking, but is not defined by either of these specific response forms (an operant is defined in terms of the dynamic consistencies in functional contingencies between classes of response and stimulus instances; see Catania, 1973). To elaborate, the procedures and the principles to which RFT appeals in explaining relational frames as operants are identical to those appealed to in explaining any operant; in particular, contacted consistencies in contingencies involving multiple exemplars. This view is reflected clearly in the following statement on generalized operants and relational frames, found in Catania’s (1998) *Learning* textbook,

. . . training with many instances may sometimes be a sufficient prerequisite for higher-order or generalized classes (e.g., training with many symmetry problems may produce generalized symmetry, training with many transitivity problems may produce generalized transitivity, and so on; such generalized classes have been called relational frames . . .). (p. 158)

Finally, as noted above in the quotations, the relational frame is defined as a unit of responding. As such, the properties of entailment and transformation of function, and the relevant contextual cues, are not separable. Any instance of relational framing, therefore, will involve entailment and transformations of function under appropriate forms of contextual control. For illustrative purposes, imagine a simple matching-to-sample (MTS) task in which a participant has been trained to match A to B and B to C, and they subsequently match C to A reliably in the absence of reinforcement. In this case, RFT assumes that particular variables will function as both Crels and Cfuncs, for establishing entailed relational responding between C and A. For example, MTS tasks are typically used in early educational exercises to establish arbitrary relations between words and pictures of objects (i.e., the MTS format may function as a Crel for coordination). Furthermore, successfully matching C to A indicates that the trained sample functions of A have been transformed into comparison functions and the trained comparison functions of C have been transformed into sample functions. The structure of the MTS task, in this case, also functions as a Cfunc because it restricts the relevant functions to choosing a comparison in the presence of a sample.

Having outlined the core features of the concept of the relational frame as described in the 2001 volume (Hayes et al., 2001), we will now summarize a relatively recent research program in RFT that has begun to highlight the importance of the theory’s field-based interbehavioral roots. Having done so, we will then revisit the core concept of the relational frame to determine the extent to which it too may align with an interbehavioral treatment (for a more complete overview of Kantorian interbehaviorism, the reader is referred to Hayes & Fryling, 2023; see also Midgley & Morris, 2006).

## Studying Relational Framing with the Implicit Relational Assessment Procedure (IRAP)

The IRAP (Barnes-Holmes et al., 2008) is an RFT-based methodology that was designed to assess the relative strength or probability of verbal relations as defined within the theory. It comprises a computerized methodology that measures response latencies to stimulus relations that are deemed either coherent or incoherent with participants' preexisting verbal histories. During each trial, a label stimulus (e.g., “flower” or “insect”) appears at the top of the screen, whereas a target stimulus (e.g., “pleasant” or “unpleasant”) appears at the center. Participants are required to select one of two response options (e.g., True vs. False) to confirm or disconfirm the label–target relation on a given trial. It is critical to note that the IRAP alternates between blocks demanding opposing responses: one block may require affirming flower–pleasant as True, whereas the next denying this relation (False). The core thesis here is that participants should tend to respond faster on trials that are coherent or consistent with their verbal histories (e.g., flowers–pleasant–True) than on trials that are not (e.g., flowers–unpleasant–True; Barnes-Holmes et al., 2010).

Performances are typically quantified by subtracting mean latencies for incoherent blocks (e.g., flower–pleasant–False) from coherent blocks (flower–pleasant–True). The resulting latency difference scores, which are typically standardized, produce four different trial-type scores. Each trial-type score reflects one of the four label-target combinations (e.g., flower–pleasant, flower–unpleasant, insect–pleasant, insect–unpleasant). These scores were initially seen as reflecting the relative “strengths” or probabilities of the four stimulus relations involved. In the history of this research, differences observed in trial-type effects were attributed largely to stimulus valence (e.g., positive and negative reactions to flowers vs. insects; see Barnes-Holmes et al., 2010). Valence-based interpretations faltered, however, when assumed neutral stimuli produced unexplained disparities in trial-type patterns. For example, Finn et al. (2018) employed the words “color” and “shape” as labels, and examples of colors (e.g., red, blue) and shapes (e.g., square, circle) as targets. The findings documented larger difference scores (i.e., faster responding during consistent versus inconsistent blocks of trials) for the color–color trial-type than for the shape–shape trial-type, despite identical response requirements (selecting True) and assumed negligible valence differences between these stimuli. This anomaly—irreconcilable with earlier models (Barnes-Holmes et al., 2010)—prompted the development of the Differential Arbitrarily Applicable Relational Responding Effects (DAARRE) model (Finn et al., 2018).

The DAARRE model attributes differential trial-type effects to the relative coherence or overlap among three specific RFT-based elements: (1) Crels or the relational properties of stimuli (e.g., the coordination between the word “color” and color words); (2) Cfuncs or the functional properties of the label and target stimuli (e.g., salience from linguistic frequency; color words occur with greater frequency in natural language than do shape words); and (3) Cfuncs or the functional properties of the response options (e.g., True may be considered generally more positive than False). With respect to the colors-and-shapes IRAP referred to above, the larger difference score observed for the color–color relative to the shape–shape trial-type may be explained as follows. In particular, assuming that colors and color words occur with great frequency in natural language than do shapes and shape words (see Keuleers et al., 2010, for lexicon database support), participants may orient more readily to the former than the latter. Thus, participants may have found it easier to respond True on color–color trials relative to shape–shape trials within coherent blocks; that is, the Cfunc properties of True overlapped, or cohered, to a greater extent with the Crel and Cfunc properties of color–color than shape–shape. Since this seminal work with shapes and colors, the DAARRE model has been used in analyzing and predicting patterns of effects that emerge on the IRAP across a range of stimuli and different participant profiles (e.g., Bortoloti et al., 2019, 2023, 2024; de Almeida et al., 2024; Finn et al., 2019; Pidgeon et al., 2021; Pinto et al., 2020; Schmidt et al., 2021). Overall, therefore, this growing body of work underscores the important role of the functional properties of stimuli as moderators of AARR.

Within the context of the current article, the important point here is that DAARRE-based research with the IRAP has emphasized the critical role of coherence among the Cfunc and Crel properties of the stimuli. As has been recently argued elsewhere, this new conceptual focus provided by the DAARRE model requires a shift in emphasis within RFT itself (see Harte & Barnes-Holmes, 2024), upon which we will now elaborate.

It is interesting that the recent shift in focus signals a revival of RFT’s foundational concerns, which initially centered on examining rule-governed behavior as complex relational networks (Hayes & Hayes, 1989), rather than prioritizing individual relational frames (Hayes, 1991). In other words, the Kantorian roots of RFT have resurfaced, particularly in how complex relational networks are now understood as dynamic fields of verbal interactants. From this perspective, the components of any relational network do not stand alone; instead, their meaning and function arise from their participation within an interactive field. For instance, the label “Color” acquires its positive orienting function in relation to the comparatively less positive function of “Shape.” Thus, the interactants actualized in a given IRAP performance collectively define the psychological event.

With this return to the interbehavioral roots of RFT, Harte and Barnes-Holmes (2024) argued that the DAARRE may be usefully interpreted through the lens of J. R. Kantor’s formula for the psychological event (Kantor, 1958). This unit of analysis (the psychological event) is distinct from more common conceptualizations in behavior analysis, and in ways that are especially relevant to conceptualizing complex behavior. We briefly summarize it here, though readers are encouraged to consult related readings for more about the psychological event or field construction (e.g., Hayes & Fryling, 2018).

The following formula summarizes the psychological event—PE = C(k, sf, rf, st, md, hi) (Kantor, 1958, p. 14). In this formula, “PE” represents the Psychological Event; “C” that all the factors constitute a single event—that is, they are an integrated whole; “k” the uniqueness of every event configuration; “sf” for stimulus function; “rf” for response function; “st” for setting conditions; “md” for medium of contact; and “hi” for interbehavioral history. We emphasize here that “C” indicates that the parts comprising the psychological event are not actually parts in the sense that they can be considered separate from the event's entirety. Rather, the parts are distinguished in this way for analytic purposes in both investigation and interpretation. The “k” term emphasizes that each psychological event is unique, in that the configuration of factors comprise an event and is not replicable. “Sf” represents stimulus function or the stimulating action of a stimulus object (note: there are important implications for the distinction between stimulus objects and stimulus functions, which we elaborate upon below). “Rf” refers to response functions that are differentiated from response forms. Just as stimulus functions are distinguished from stimulus objects, response functions are distinguished from responding organisms. “St” stands for setting conditions, which are features of the circumstances in which stimulus and response functions are taking place. It is important to note that setting factors include environing and organismic circumstances. Examples of the latter sort include such conditions as fatigue, illness, hunger, intoxication, and so on (e.g., Kantor, 1958; Kantor & Smith, 1975). The setting also includes other ongoing interactions as when a bilingual person speaks the same language as is being spoken by other persons in a particular circumstance. “Hi” refers to interbehavioral history, underscoring the premise that a person’s history is a cumulative presence in all ongoing psychological events (Hayes, 1992a). An important point in this regard is the nonrepetition of psychological events; that is, each event has a history that includes a previous event that the previous event does not have. For analytical purposes, interbehavioral history may be described as being made up of two elements, namely stimulus evolution and reactional biography (just as stimulus functions and response functions are distinguished for analytic purposes). Finally, “md” refers to medium of contact, which refers to factors that permit the contact of organisms with stimulus objects (e.g., light being the medium of contact for a visual stimulus object).

### The DAARRE Model: A Field-Based Interpretation

Kantor’s framework posits that the Psychological Event (PE) is synonymous with the entire field of contributing factors (C). In this context, the PE represents the complete IRAP performance analyzed through the DAARRE model. The C element underscores that all elements of the model form an indivisible whole—removing any single factor would constitute a completely separate and novel event (i.e., removing a factor would not be seen as causally influencing a change in the same original event). For instance, eliminating the shape stimulus (and its less positive function) would inherently redefine the more positive function of the color stimulus, as these comparative functions co-define each other within the model. The inclusion of the k factor further emphasizes that the DAARRE model captures a distinct psychological event, allowing for individualized interpretations of IRAP performances. Thus, even repeated administrations of the same IRAP to one person may yield unique psychological events each time.

The DAARRE model explicitly differentiates between stimulus objects (sf) and their functions, as well as between response functions (rf) and the individuals producing them. For example, although color and shape exist as independent stimulus objects, their relatively positive (and less positive) functions only actualize within the model. Likewise, selecting "True" or "False" constitutes an individual’s response, but the coherence (or lack thereof) of these responses with other model elements (e.g., Crel properties) is only actualized within the DAARRE model.

Kantor’s formula also incorporates setting factors (st), which influence how stimuli function within the IRAP. For example, environmental conditions, such as temperature, may shift the Cfunc properties of certain stimuli—cold drinks might acquire a relatively positive evoking function in a hot setting, whereas hot drinks could do so in a cold setting. Beyond physical conditions, setting factors encompass broader contextual variables, including instructional effects (Finn et al., 2018) and cultural influences. For example, Power et al. (2017) found divergent IRAP performances between white Irish participants and Black African immigrants, possibly due to language barriers or the social dynamics of completing the task in a predominantly white setting. This highlights the expansive scope of setting factors in field-based analyses, which resist reduction to simplistic experimental manipulations.

It is critical to note that a field-based approach guards against reifying mentalistic constructs (e.g., "racial bias") as fixed, internal causes of behavior. Traditional psychology often treats behavioral measures as proxies for such constructs, but an interbehavioral perspective rejects this, viewing each event as a unique, dynamic interaction. Terms, such as Crel and Cfunc, are not invoked as mediating variables but as descriptors of relational patterns within the field. An IRAP score does not contain these properties—rather, their influence emerges from the interaction among trial-types.

The medium of contact (md) in most IRAPs has been visual–tactile, but the DAARRE model has recently been extended to other modalities, such as auditory–visual presentations (Parreiras et al., 2025). Although this may seem minor, it illustrates the model’s flexibility in accommodating diverse interbehavioral contexts.

Finally, interbehavioral history (hi) is integral to the DAARRE model, because stimulus functions are shaped by prior learning. For instance, the relative dominance of color over shape in typical IRAPs may reflect the higher frequency of color-related language in everyday experience. However, this history does not *cause* performance in a linear fashion—it is embedded within the model as part of the interactive field. If an architect, for whom shape may hold greater historical significance, exhibited reversed dominance (shape over color), the model would prompt an investigation into such unique histories. Even immediate preexperimental factors, such as instructions or prior task exposure (Finn et al., 2016, 2018), contribute to the interbehavioral field, emphasizing that no element operates in isolation.

Although the shapes-and-colors example may appear simplistic, it underscores a crucial point: history is not a detached precursor but a constitutive aspect of the psychological event. By framing the DAARRE model as a field of co-defining interactants, researchers may be better equipped to explore the dynamic, contextually embedded nature of AARR.

Before continuing, it seems important to clarify that the field-theoretical analysis provided above applies to the IRAP performance as a whole—it cannot be reduced meaningfully to the examination of isolated responses. In other words, the psychological event is the complete IRAP performance, and the complete IRAP performance is the psychological event, thus entailing a deeply historical analysis. At the same time, we must acknowledge that the stimulus and response functions operating on any single IRAP trial are transient. Prior instances of these functions become part of the contextual backdrop against which new functions emerge. In this sense, the IRAP performance unfolds as a dynamic event: each trial’s responses occur within a shifting context, with every subsequent trial carrying a slightly richer history than the one before. Viewed through this lens, a more granular analysis of IRAP responding could certainly be pursued—but for different analytical purposes than those guiding the present field-based interpretation.

## Back to Basics: An Interbehavioral Interpretation of the Concept of the Relational Frame

Having explored how the DAARRE model may be interpreted through the lens of an interbehavioral psychological event, an obvious and important question quickly emerges. In particular, will the core foundational concepts of RFT also yield to a full interbehavioral analysis? If they do not do so, this could be seen as rendering the theory somewhat incoherent. That is, if all features or aspects of RFT do not yield to an interbehavioral analysis, it could be argued that RFT no longer represents a single overarching behavioral account of human language and cognition. It thus seems important to determine if the interbehavioral analysis of relational complexity, as outlined thus far in the context of the DAARRE model, could also be applied readily to more foundational concepts in RFT. To begin to address this question, the remainder of the current article will focus on an interbehavioral reappraisal of the core concept of the relational frame itself.

### The Relational Frame: An Interbehavioral Reappraisal

As noted previously, the concept of the relational frame refers to a class of AARR involving contextual control of mutual and combinatorial entailment, and transformations of stimulus functions. The specific behavioral patterns of entailment and transformation of function are determined by contextual cues. The properties of these cues are established through an extended history of learning, through interactions with the verbal community, to respond appropriately in the presence of those cues (i.e., the cues determine the relational responding rather than just the formal properties of the stimuli or relations involved). In reappraising the concept of the relational frame through an interbehavioral lens, we will first review the interbehavioral concepts of stimulus and response functions, including substitute stimulation and responding, setting factors, and interbehavioral history. We focus on these concepts in particular because they seem most pertinent to our current objective. That is, we will explore the extent to which these concepts allow for an interbehavioral analysis of the core concept of the relational frame.

### Stimulation and Responding

Whereas more common constructs in behavioral psychology involve linear sequences of discrete events of some variety (e.g., S → R / respondent, Sd → Bx → Sr / operant), the psychological event of interbehavioral psychology does not.^2^ Indeed, the focal point of the psychological event is the function of stimulating and responding (Sf ←  → Rf), the implication being that there is no stimulation without responding, no responding without stimulation.^3^

Interbehavioral psychology emphasizes the distinction between stimuli as objects and as functions, as well as that between responses as biological forms and functions. This is to say, as psychologists, we are interested in the actions of stimuli and responses—their functions, not their sources. The term “function” is herein used in a purely descriptive sense. Uses of this term that imply cause, purpose, or reason are out of keeping with interbehavioral philosophy. As the element “C” in the formula for a psychological event indicates, the factors comprising an event field are not actually parts in the sense that they can be considered separate from the event's entirety. The psychological event is, rather, conceptualized as an integrated whole in which no abstracted part can be held to be causally responsible for any other part or for the entire whole of which it is itself a part. In essence, this means that stimuli are not causally responsible for behavior in interbehavioral psychology. This is in stark contrast to common uses of the term “function” in behavioral circles (e.g., Fryling & Hayes, 2011; Kantor, 1950, pp. 156–157).

It is important to note that the distinction between stimuli as objects and stimuli as functions, as well as that between response forms and psychological responding, permits an analysis of how stimulus objects might develop the psychological properties of other stimulus objects, and how these psychological properties might be present in the absence of those objects. In the interbehavioral perspective, this is referred to as the development of substitute stimulation—and the corresponding response as a substitute or implicit response (e.g., Kantor, 1924/1926). We next describe an example to highlight this important concept and process.

An individual might interact with a picture by noticing the colors or textures involved, perhaps it is also especially clear or blurry. These are responses to the object properties of the picture as a stimulus. But in addition to these properties, one might also respond with respect to the picture, let us say of a lake, by seeing other lakes they have visited in the past, the people they were there with, the activities they engaged in, and much more (e.g., if it was a cold day at the lake, the individual might respond as if they were now cold by pulling their jacket close, and saying “I remember how cold it was that day”). Indeed, reading this text alone may actualize what are referred to in interbehavioral psychology as substitute stimulus functions for the reader (e.g., reminding the reader of a particular day they spent at a lake). These stimulus properties, and corresponding responses (Sf←→Rf), have nothing to do with the physical features of the picture. They have nothing to do with the colors or texture of the picture or the extent to which the picture is clear or blurry (the irrelevance of these features *alone* for the psychological event may be illustrated by considering how another person with a different history might respond to the picture). The distinction between a stimulus as a physical object and as a stimulus function is thus key to understanding complex psychological happenings from an interbehavioral perspective (along with corresponding response functions, as stimulation always occurs as a functional relation; Sf←→Rf).

Kantor proposes that substitutional stimulation comes about under two sets of circumstances, both of which have been identified and acknowledged by others. The first of these, known elsewhere as generalization, occurs when sources of stimulation share properties, primarily of a formal sort. This is to say, responding originally occurring with respect to stimulation inhering in one source may come to occur with respect to stimulation operating from another formally similar nonoriginal source. We respond to a chair as we did to a previously encountered chair based on their formal similarities. Happenings of this sort are not restricted to shared *formal* properties in persons with linguistic repertoires (we will return to this later).

A second set of conditions under which substitute stimulation may develop is one in which original and nonoriginal sources are present in spatiotemporal arrangement such that responding with respect to stimulation in both sources is ongoing. These circumstances are such that stimulation originally inhering in one of these sources may come to inhere substitutionally in the other, and vice versa. Kantor (e.g., 1924/1926) describes how a history of contact with association conditions can result in a stimulus object developing the substitute stimulus properties of another object. It is important to remember that association conditions are not mentalistic happenings in interbehavioral psychology—individuals do not engage in association nor are they said to be *associating.* Association conditions describe the co-occurrence of two or more things *in space and time*. They may involve stimuli and stimuli, stimuli and responses, responses and responses, stimuli and settings, and more (e.g., Kantor, 1924/1926). The important feature is that there is a co-occurrence of things or events in space and time. Moreover, a history of co-occurrence alone is not enough—an individual must *respond* with respect to these association conditions in some way. For instance, sitting at a particular cafe in which a particular sort of music is played (an association between the cafe and music) may result in the person seeing the cafe when just hearing the music, but *only* because the individual has a history of being in the cafe while hearing the music. At the same time, although that individual was sitting at a particular cafe listening to music, there were also other cafes nearby where other music is playing. When the person later hears the music that was playing at the cafe they were not present at, they will not see the cafe, because they were not there/have no history of responding with respect to the association condition in any way. In this example, when the person sees the cafe while hearing the music we would say that the music is substituting for the cafe (or has developed the substitute stimulus functions of the cafe).

This second set of conditions overlaps to some extent with the traditional concept of Pavlovian conditioning, including higher order conditioning and sensory preconditioning, except that it is held to be pervasive, multiplicative, and without directionality. Because the conditions for substitute stimulation of this sort are always present, the actualization of substitute stimulus functions is always ongoing. Further, once substitute stimulation comes to inhere in a nonoriginal source, its operation from that source affords additional opportunity for substitution. Seeing by way of hearing, as in the example of seeing a person’s face when only the sound of their voice is present, affords the development of substitute stimulation originally inhering in the person’s face as a source. That is, one might not only see a face when a previously recorded voice is present, but also respond to the substitute stimulation inhering in the face as a source (e.g., feeling grief if, for example, the person is deceased). This is a case of substitution upon substitution, or “double substitution” as it is sometimes called (e.g., Muñoz-Blanco & Hayes, 2017). Substitution upon substitution, like first-order substitution, may afford additional substitutions (e.g., grief affording guilt about a previous negative interaction with the deceased individual). At this point, it is worth noting that we assume that substitute stimulation as conceptualized here is not wholly absent in nonlinguistic organisms. However, we would argue that it is greatly facilitated by human language and so enormously amplified by it that the repertoires of linguistic and nonlinguistic organisms can scarcely be compared.

It goes without saying that substitute stimulus←→response functions of this sort are features of most psychological interactions. Kantor refers to a field in which substitution is occurring as an implicit^4^ field (e.g., Kantor, 1924/1926). As noted above, language appears to make substitution more pervasive, as, for example, when specific sounds (i.e., words) begin to occur in association with other environing things and events.

#### Stimulus Substitution and RFT

It is important to note that there appears to be some overlap between the concept of stimulus substitution and the *relational* part of RFT. That is, relating in RFT is defined as responding to one thing or event in terms of another, which seems broadly consistent with the idea of the substitution of stimulus functions (also Hayes, 1992b). In response, one might argue that there may be overlap between the concept of substitution and that of transformation of functions specific to the frame of coordination or equivalence (i.e., where a similar function is observed for two separate stimuli). However, RFT incorporates, and is indeed defined in part by, different patterns of relational responding (i.e., frames). The question remains, to what extent the interbehavioral concept of stimulus substitution may also address, in a meaningful way, the RFT idea of multiple relational frames (i.e., frames other than coordination).

The concept of substitution in interbehavioral psychology may readily accommodate multiple relational frames if a stimulus–response function can itself involve a formal relationship between or among stimuli. For example, consider the physical difference in size between a tennis ball and a basketball. Of course, the two balls may have separate stimulus–response functions, but in principle, the physical relationship between the two stimulus objects may also inhere a stimulus–response function. That is, relations among association conditions (e.g., smaller than, larger than) may themselves be considered stimulus objects (i.e., a source of stimulation) with the potential to harbor stimulus functions. Responding with respect to such a stimulus object would not necessarily require a linguistic repertoire, because nonhumans readily respond in accordance with such formal relations (e.g., a cockroach will approach the darker of two areas). Nevertheless, it could be argued that responding with respect to such a stimulus object (a nonarbitrary formal relation) may afford stimulus–response functions with respect to linguistic or arbitrary relations. For example, once a stimulus–response function has been established for stimuli that differ formally in size (e.g., bigger/smaller than), that source of stimulation may develop substitute stimulus functions for stimuli that differ *arbitrarily* in size (e.g., X is larger than Y). Or more colloquially, the organism is responding to X and Y *as if* X was a basketball and Y was a tennis ball. Why and under what circumstances this occurs can only be explained by looking to other participants within the interbehavioral field; in particular, history and setting factors, to which we now turn.

### Setting and History

As previously discussed, Kantor (1958) construed psychological events as fields of integrated factors centered around functions of stimulating and responding. The function of stimulating and responding is the focus of the event field in the sense that a given psychological event is identified by the specification of this function. Surrounding the focal function, or more precisely, the immediate circumstances in which the focal function is taking place, is the setting in Kantor’s formulation. In what follows, these factors, along with the nature of their participation, and their significance for an analysis of the interbehavioral histories of individuals, are described.

#### The Setting

Setting conditions are factors of several types. As they are commonly imagined, they are environing factors of a rather broad or encompassing sort such as ambient temperature or weather conditions such as rainfall. They may also be construed as compounds or organizations of circumstantial elements as when a particular place, such as a room or a city, is identified as the setting. Also included in this category are stimulus objects and events of a more discrete sort (e.g., a motorcycle in the broader setting of a garage). By extension, it might seem that the setting amounts to absolutely everything in the immediate environs when a function of stimulating and responding is taking place. This is not how we have come to understand setting conditions, however. Rather, what constitutes the setting for a given individual’s interbehavior is a matter of any element that *participates in* that psychological event as an interactant with the stimulus response functions (i.e., a setting condition is not simply everything in the environment including nonparticipating events). For example, during a conversational interaction, the sound of an airplane flying overhead may serve as a setting factor in terms of “disrupting” the conversational flow (we return to this argument below).

Still further, setting conditions are not limited to environing factors. As mentioned above, they also include bodily conditions such as fatigue, pain, intoxication, and deprivations of various sorts. Finally, other ongoing interactions, particularly of a cultural sort where alternative repertoires are available, may constitute setting conditions. For example, being present in a circumstance where a particular language is spoken may serve as a setting condition (Kantor, 1977).

Amid these many factors and their organization, functions of stimulating and responding are taking place. Given that stimulus objects and organisms are sources of multiple stimulus and response functions, the question arises as to what accounts for the function that takes place at a given time. It may be tempting to suggest that the focal functions are actualized by the setting in the sense that it hinders or facilitates the occurrence of some functions over others when alternatives are possible. However, the identification of the psychological event as an integrated field cautions against this interpretation. Indeed, in keeping with a field perspective, the setting cannot be held to influence the psychological event of which it is, itself, an integrated factor. To put it another way, the setting is not a stand-alone entity capable of exerting influence over the factors participating in a psychological event field. It is itself a participating factor in that field. As the function of stimulating and responding constituting the focus of an event field is changing, so too is the setting. The setting changes as different collections of factors obtain in new arrangements—each configuration constituting a new event field. Staying with our previous example, the sound of a plane might be a setting condition, participating in an event field in which a conversational interaction is taking place. Still, the sound does not hinder or facilitate the stimulus–response functions that constitute the conversation *as a psychological event*. Rather, it is a part of the event itself. In common sense terms, the sound of the plane may be seen as “hindering” the ability of a listener to hear what a speaker says, but in terms of the psychological event, what is heard is heard (i.e., the listener does not hear something perfectly that is subsequently hindered in some way).

#### Interbehavioral History

Accounting for an organism’s history has always been a challenge in the discipline of psychology and the various solutions to this problem have not been particularly helpful (Hayes, 1992a). Interbehavioral history is conceptualized for analytical purposes as involving two elements: reactional biography and stimulus evolution (e.g., Kantor, 1924/1926; Kantor & Smith, 1975). These elements represent the historical developments of response and stimulus functions, respectively. The present circumstance may be understood as the points to which these elements have evolved independently and in relation to one another (Hayes, 1992a). Examples of the development of these elements of an individual’s history may be helpful. A gradual change in the formal characteristics of a response to the physical properties of a stimulus object (e.g., how a child comes to hold a cup), such as to render it more refined, better suited, or more effective in some way, exemplifies the development of the reactional biography. The element of stimulus evolution may be exemplified by considering the lifelong development of the substitutional functions of stimulus objects. Stimulus objects initially harboring stimulus functions, having their sources only in their physical properties, subsequently come to harbor the stimulus functions of other objects by virtue of their temporal and spatial relations with those objects; these, in turn, then afford the development of additional substitutional properties.

As mentioned, these elements of interbehavioral history are distinguished to permit emphasis on one or the other in the analysis. However, like the stimulus and response elements of a psychological function, the elements of stimulus evolution and reactional biography are conceptualized as features of a unity. There are no responses without stimuli, no stimuli without responses, and no history that has not involved their interactions. However, a history of interactions is not, itself, a factor localizable in a *presently occurring* event field. What is to be found in present circumstances are the points to which these elements have evolved in relation to one another (Hayes, 1992a). The functions of stimulating and responding in immediate circumstances reveal these points.

#### Setting, History, and Multiple Stimulus Relations

Having now outlined setting and history we are ready to employ those concepts in explaining how a stimulus–response function that has been established for stimuli that differ formally in size (e.g., a basketball is larger than a tennis ball), may develop substitute stimulus functions for stimuli that differ *arbitrarily* in size (e.g., X is larger than Y). According to RFT, contextual cues, such as the Crels “larger than” or “smaller than,” determine the appropriate relational responses to stimuli that differ in nonarbitrary ways (as noted earlier, Cfuncs determine the dimensions along which functions transform). Across many exemplars, these cues also occur in the presence of stimuli that are not related in any clear nonarbitrary manner, and thus an individual may respond to X as larger than Y having been told that Y is smaller than X. From an interbehavioral perspective, such contextual cues are interpreted as setting factors that operate as such given the interbehavioral history in which those cues have participated. The stimulus–response functions involved in a “relational response” with regard to X and Y, along the dimension of size, occur in the wider interbehavioral context of setting factors and history. In contrast to the way in which discussion within RFT often affirms history and cues as *determining* or *controlling* relational responses, an interbehavioral interpretation views them as participants in an integrated field. This is a difference between the two ways of conceptualizing relational frames. The fundamental difference is in attributing causal influence to cues in one case and not the other.

In noting the foregoing contrast, it is worth emphasizing that, strictly speaking, contextual cues (and their history) in RFT have never been defined as “existing” outside the concept of the relational frame as functionally defined. As noted earlier, the concept of the relational frame is defined as a behavioral unit of responding in which the properties of entailment and transformation of function, and the relevant contextual cues, are *not separable*. In other words, the metaphorical concept of the frame in RFT is most usefully interpreted as the contextual cues in any instance of relational responding (i.e., the contextual cues “frame” the relational response). From this perspective, the concept of the frame by definition *includes* the cues as integrated into the relational response (i.e., they are co-defining, therefore very much aligned with an interbehavioral view).

### Summary Statement

At this point we have argued that the concept of the relational frame may be readily interpreted using the interbehavioral concepts of stimulus substitution, stimulus–response functions, setting, and history. In particular, this first involves acknowledging that a stimulus–response function can itself involve a formal relationship between or among stimuli. As noted earlier, two balls may have separate stimulus–response functions, but in principle, the physical relationship between the two may also inhere a stimulus–response function. Furthermore, once a stimulus–response function has been established for stimuli that differ formally in size, that source of stimulation may develop *substitute* stimulus functions for stimuli that differ *arbitrarily* in size. Finally, we argued that this substitution operates in the wider context of a particular organization of setting factors and interbehavioral history (i.e., Crel and Cfunc contextual cues in RFT).

In our view, therefore, the core concept in RFT (i.e., the relational frame) appears to yield readily to a field-based interbehavioral interpretation. Of course, much more remains to be done in that there is more to RFT than just the concept of the relational frame. Indeed, recent developments in the theory have presented a hyper-dimensional, multi-level (HDML) framework for conceptualizing much of the detail within the theory (Barnes-Holmes & Harte, 2022; Barnes-Holmes et al., 2020), but a treatment of this is beyond the scope of the current article (but see Harte & Barnes-Holmes, 2024, for recent initial efforts in this regard). In any case, before closing, it seems wise to consider potential objections to the core thesis of the current article. In doing so, we will return to the seminal text on RFT (Hayes et al., 2001) in which specific objections to an interbehavioral approach were articulated. In particular, we will consider these objections to determine if they still stand in light of the current analysis. Before continuing, the reader should note that the responses we will present in the next section are largely the result of the ongoing empirical and conceptual efforts in RFT that have emerged in the past number of years. However, that is not to say there were no legitimate responses to Hayes et al.’s criticisms at the time of the initial publication in 2001. We direct the reader to Appendix A for brief responses to each of those criticisms that also applied at the time of the original publication, whereas we will focus on the current responses within the main body of the text.

## Initial RFT Objections to Kantor’s Interbehaviorism

Although RFT is presented in the 2001 treatise as having a “... slightly Kantorian feel” due to the influence of L. J. Hayes (Hayes, 2001, viii), in chapter 1 of that volume, S. C. Hayes, Blackledge et al. (2001) present three core objections to Kantor’s interbehaviorism. First, they state that,

The single biggest problem is that Kantor’s system did not lead to a robust empirical tradition of language research. Kantor was a philosophical psychologist, not an empirical scientist. His work in general was difficult to translate into specific experimental preparations. (p. 8)

We would agree that when RFT first emerged in the mid-1980s, analyses of the type of phenomena in which RFT was interested were fairly scant within behavior analysis generally—in this sense, 2001 RFT could be seen as a major contribution. Irrespective of one’s view of RFT, that initial theory certainly led to a rich vein of research on human language and cognition. It is critical to note, however, that research has become increasingly complex so as to occasion the current reconsideration of the theory through the lens of interbehaviorism. With this in mind, and as outlined recently elsewhere (Harte & Barnes-Holmes, 2024), RFT-based experimental analyses over the past number of years have increasingly drawn on field-theoretical analyses and concepts. In particular, experimental research that has sought to analyze complex relational networks using the IRAP has increasingly involved conceptualizing responding on the procedure as involving a field of behavioral (verbal) interactants (e.g., Barnes-Holmes, et al., 2020; Bortoloti et al., 2023; Harte et al., 2023; Pidgeon et al., 2021; Pinto et al., 2020; Schmidt, et al., 2021). Indeed, Harte and Barnes-Holmes (2024) have recently interpreted responding on the IRAP in terms of Kantor’s formula for a psychological event and argued that doing so readily invites rich conceptual and empirical questions. It seems, therefore, that the first objection raised by Hayes et al. (2001) could be questioned because, in simple terms, some experimental analyses appear to be bringing RFT back to Kantorian interbehaviorism (see also Finn & Barnes-Holmes, 2021).

Second, Hayes, Blackledge et al. (2001) argued that,

. . . to do an experiment one has to begin to emphasize one aspect of the field over others (as when one makes a distinction between independent and dependent variables, for example), but in Kantor’s system all participants in a field are equal. (p. 8)

Of course, we agree that one cannot study “the integrated field”—you can only study parts of it and thus all investigation will seem limited in this way. Indeed, the point of any experiment, operant or interbehavioral, is to simplify in some respect the complexities of behavior–environment interactions occurring outside of the laboratory. Thus, we would argue that the statement above is valid, but this should not involve dismissing the value of philosophy and theory in the first place. In this light, experiments certainly involve identifying controlling and controlled variables, and the interbehavioral field concept at first blush does not seem to align well with this experimental focus. We would argue, however, that clearly distinguishing between investigative and interpretive analyses/constructs readily solves this issue (e.g., Kantor, 1957; Smith, 2007). In particular, although it may be useful for investigative purposes to target one specific element of the event field, and even employ a linear approach in planning an experimental sequence, such activities should not be confused with the interpretive constructs of a purely field-based analysis. Consider, for example, the experimental work referred to in the previous point. As outlined by Harte and Barnes-Holmes (2024), linear analyses and contingency-based constructs were employed in much of this experimental work, and indeed allowed many analytic goals to be achieved. However, relatively sophisticated functional-analytic explanations of the complex patterns of behavior emerged only when those behaviors were interpreted within a field-theoretic light (see Harte & Barnes-Holmes, 2024, for further detail). As such, there does not seem to be any issue with focusing on one element of the interbehavioral field for investigative purposes while recognizing that interpretively all other elements of the field are of equal importance and involved in any interpretive analysis. In making this argument, we are suggesting that investigative and interpretive analyses should cohere, but one should not be confused or conflated with the other. In any case, given the foregoing, the second objection raised by Hayes et al. (2001) could also be seriously questioned.

Finally, Hayes, Blackledge et al. (2001) stated,

The other empirical difficulty comes from the limited set of behavioral principles involved. Kantor’s reliance on formal similarity and a loose form of associationism simply did not yield coherent experimental procedures and the processes Kantor supposed would account for the substitutive functions of language did not translate readily to the laboratory. (p. 8)

As we have argued in the current article, permitting for stimulus substitution for stimuli that differ arbitrarily opens the door for the entire RFT research program to yield (at least potentially) to interbehavioral field-based analyses. In particular, Kantor’s emphasis on formal similarity and associationism appear to be garnered with a greater level of precision in the context of RFT. Furthermore, irrespective of one’s view of RFT as a theoretical account, one cannot deny that it has served to generate a relatively rich and ongoing program of experimental and applied research. If this research and its future development is increasingly viewed from an interbehavioral perspective, the above criticism concerning the absence of coherent experimental procedures and processes appears to fade away.

## Conclusion

In closing, we recognize that the conceptual integration of the core concept of the relational frame in RFT with Kantorian interbehavioral theorizing may appear to challenge the emphasis on operant-based analyses within the RFT account. Although this is definitely the case from an interpretive perspective, investigative analyses will almost certainly continue to benefit from, and indeed be guided by, some level of operant conceptualization. The psychological domain of human language and cognition is without doubt highly complex and this complexity seems to be reflected readily in field-based theorizing. However, when pursuing the investigative goals of prediction-and-influence, it seems necessary to parse out the event field in some manner or other, but in doing so always remaining aware that the complex has been simplified temporarily in the service of achieving some specific investigative goal. We hope what we have offered here provides some assistance in walking this conceptual tightrope.

Finally, it seems important to ask why we should attempt to walk this tightrope in the first place? For instance, we imagine that at least some RFT researchers will balk at what we are offering here, arguing that RFT is doing just fine as it is, and that the foregoing analysis is not needed. Leaving aside that RFT is historically rooted in the Kantorian tradition, and on the grounds of intellectual honesty this should not be denied, we believe there could be clear benefits to pursuing an interbehavioral approach to RFT. Apart from encouraging RFT researchers to address the sheer complexity of human language and cognition, which in our view is more clearly revealed through an interbehavioral lens, it also provides a strong prophylactic against slipping into mentalistic interpretations of RFT. Indeed, it is easy to see how such interpretations may arise when relating stimuli is sometimes described in the literature as something an organism *simply does* (i.e., rather than as a historically situated pattern of behavior with respect to those stimuli). Although “doing” is not explicitly mentalistic in a mainstream cognitive sense (i.e., mental processing), it readily attributes "causal" agency to the organism (i.e., the doing of something). And when this “doing” is "relating," particularly if the relating is deemed a private event, the slippery slope into full blown mentalism is difficult to avoid (see Hayes & Fryling, 2009; see also Baum, 2011a, b, and Rachlin, 2011) . If, however, relational responding is clearly defined as a stimulus–response function, loose language (in the service of easy communication) is less likely to be interpreted as blatant examples of mentalism. Furthermore, it is our hope that going back to the interbehavioral foundations of RFT may facilitate further development of the work as it continues towards a more comprehensive understanding of the important phenomenon that is human language and cognition.

## Data Availability

Not applicable.

## Appendix A

### Initial RFT Objections and Responses to Kantor’s Interbehaviorism^5^

1. The single biggest problem is that Kantor’s system did not lead to a robust empirical tradition of language research. Kantor was a philosophical psychologist, not an empirical scientist. His work in general was difficult to translate into specific experimental preparations (Hayes, Blackledge et al., 2001, p. 8).

**Response**: Kantor was also a scientific psychologist. As Kantor (1953) would put it, experiments are not magic. They are investigations of partial happenings—happenings characterized by limited sets of properties and simple metrics, taking place in odd and highly routinized circumstances. In other words, experimental preparations do not equate to the linguistic event field. Added to this, the results of given experiments can be interpreted in different ways—and the interpretations matter (as least as much as research designs.)

2. to do an experiment one has to begin to emphasize one aspect of the field over others (as when one makes a distinction between independent and dependent variables, for example), but in Kantor’s system all participants in a field are equal (Hayes, Blackledge et al., 2001, p. 8).

**Response**: This argument is not about how it is impossible to study all things. No one studies all things. What is studied depends on the interests of investigators. The argument seems to be an objection to the absence of causality in the system. The objection is about how it is necessary to pitch some events—namely, causes (influencers, controlling variables, independent variables) against others, namely effects (dependent variables) in order to achieve an understanding of those events. It is important to remember that causality is a premise (of an outdated philosophy), not an event (Hayes et al., 1997). Further, empirical research being discussed involves manipulating one event to see what happens to another event. This is what an interbehaviorist would do as well: manipulate setting factors and see how the field reconfigures.

3. The other empirical difficulty comes from the limited set of behavioral principles involved. Kantor’s reliance on formal similarity and a loose form of associationism simply did not yield coherent experimental procedures and the processes Kantor supposed would account for the substitutive functions of language did not translate readily to the laboratory (Hayes, Blackledge et al., 2001, p. 8).

**Response**: It is odd that interbehavioral psychology would be said to have a limited set of principles when it could be argued that operant psychology, almost by definition, is very much focused on one core principle: reinforcement. It might seem as if there are other principles (e.g., stimulus control, punishment, extinction) but all of these appear to be derived from the one principle of reinforcement. What always seems to be the subject of investigation in operant psychology, as well as in RFT, are relations of stimulating and responding. This is what interbehavioral psychology is all about as well.

## Footnotes

1 It is important to acknowledge at this point that the term “functional” has been used in a number of different ways within behavior analysis (e.g., Fryling & Hayes, 2011). Sometimes, it is used in a way that is broadly similar with its common-sense usage; that is, the use to which something can be put. For example, the function of a screwdriver is to tighten or loosen screws. Other times it is used in an explicitly causal manner when discussing the impact a particular stimulus may have on responding; for example, when saying a rat’s lever pressing increased as a function of the delivery of food pellets (inferring that the contingent delivery of food pellets *caused* an increase in responding). In other cases, the term has been used to refer to a correlation or co-variation between two separate events without any explicit causal inference. This overlaps somewhat with the previous example but without inferring any sort of causal relationship between the events; for example, observing that lever pressing only increases when followed by food pellets may establish a functional relation but not an explicitly causal one. Finally, the term “function” has been used within interbehavioral psychology to indicate a relational unit (sf←→rf), similar to the way in which a line is described as a function within mathematics (Kantor, 1950). This is broadly similar to the noncausal use of the term “function” as outlined in the previous example. However, in interbehavioral thinking, the functional relation is not between separate events, but defines those events as a unit. In other words, the function *is* the unity of those two events, and causality is thus completely excluded (we will return to this specific use of the term function later).

2 In arguing that common behavioral constructs involve linear sequences of discrete events, we are not suggesting that the relevant units of analysis are *necessarily* linear. For example, the operant contingency must include: (1) If Sd and if Bx, then Sr; (2) if Sd and if no Bx, then no Sr; and (3) if no Sd and if Bx, then no Sr. In conceptual terms, all dimensions of the contingency must hold simultaneously; if any dimension is absent, there is no contingency. Furthermore, an operant contingency may be considered cyclical (i.e., nonlinear) in the sense that the occurrence of a reinforcer is seen as “feeding back” to both the discriminative stimulus and response classes (i.e., the occurrence of the reinforcer increases the future probability of responding in the presence of the discriminative stimulus and reduces that probability in the absence of the stimulus). Nevertheless, if an operant contingency is conceptualized as involving separate or discrete psychological events, this may be contrasted with the interbehavioral construct of the psychological event in which there is only ever one unfolding event. If, however, an organism’s contact with an operant contingency, at any point in time, is interpreted as a single unfolding psychological event (which includes the history of previous contacts with that contingency but with no causal inferences), this would render it a more field-like, albeit not a strictly Kantorian conceptualization. We suspect, however, that few behavior analysts conceptualize the operant in this latter way.

3 Kantor was specific on the stimulus and response being a single phenomenon throughout his career. For example, in 1933 he stated, “While it is customary to think of a stimulus and a response conception, there really is a single phenomenon involved” (Kantor, 1933, p. 325), and in 1977 he stated, “But when the behavior is uniquely psychological the actions of the organisms or persons are highly integrated, and the notion that a stimulus is something to make the organism act fades completely away” (Observer, p. 505). These examples indicate the extent to which Kantor approached stimulation and responding in a unique way.

4 Note that Kantor’s use of the term “implicit” should not be confused with its more recent use in other areas of psychology, such as in so-called implicit cognition or attitudes.

## References

[CR1] Barnes-Holmes, D., Barnes-Holmes, Y., & McEnteggart, C. (2020). Updating RFT (more field than frame) and its implications for process-based therapy. *The Psychological Record,**70*, 605–624. 10.1007/s40732-019-00372-3

[CR2] Barnes-Holmes, D., Barnes-Holmes, Y., Stewart, I., & Boles, S. (2010). A sketch of the implicit relational assessment procedure (IRAP) and the relational elaboration and coherence (REC) model. *The Psychological Record,**60*(3), 527–542. 10.1007/BF03395726

[CR3] Barnes-Holmes, D., & Harte, C. (2022). Relational frame theory 20 years on: The Odysseus voyage and beyond. *Journal of the Experimental Analysis of Behavior,**17*(2), 240–256. 10.1002/jeab.733 10.1002/jeab.733 35014700

[CR4] Barnes-Holmes, D., Hayden, E., Barnes-Holmes, Y., & Stewart, I. (2008). The implicit relational assessment procedure (IRAP) as a response-time and event-related-potentials methodology for testing natural verbal relations: A preliminary study. *The Psychological Record,**58*(4), 497–516. 10.1007/BF03395634

[CR5] Baum, W. M. (2011a). Behaviorism, private events, and the molar view of behavior. *The Behavior Analyst,**34*(2), 185–200. 10.1007/BF03392249 22532740 10.1007/BF03392249 PMC3211378

[CR6] Baum, W. M. (2011b). No need for private events in a science of behavior: Response to commentaries. *The Behavior Analyst,**34*(2), 237–244. 10.1007/BF03392255 22532746 10.1007/BF03392255 PMC3211384

[CR7] Bortoloti, R., de Almeida, R. V., de Almeida, J. H., & de Rose, J. C. (2019). Emotional faces in symbolic relations: A happiness superiority effect involving the equivalence paradigm. *Frontiers in Psychology,**10*, 1–12. 10.3389/fpsyg.2019.00954 31114529 10.3389/fpsyg.2019.00954 PMC6503112

[CR8] Bortoloti, R., de Azevedo, A.P.R.A., Harte, C., & Barnes-Holmes (2024)*.* Predicting and interpreting patterns of responding on the IRAP in the context of facial emotions and depression. *The Psychological Record*, *74*(3), 291–300. 10.1007/s40732-024-00607-y

[CR9] Bortoloti, R., Schmidt, M., Harte, C., & Barnes-Holmes, D. (2023). Feel the func: Interpreting IRAP performances based on Cfunc versus Crel stimulus properties. *The Psychological Record,**73*, 363–373. 10.1007/s40732-023-00557-x

[CR10] Catania, A. C. (1973). The concept of the operant in the analysis of behavior. *Behaviorism,**1*(2), 103–116.

[CR11] Catania, A. C. (1998). *Learning* (4th ed). Prentice-Hall.

[CR12] de Almeida, R. V., Barnes-Holmes, D., & Leslie, J. C. (2024). Differential trial-type effects in an implicit relational assessment procedure: Extending the DAARRE model. *The Psychological Record,**74*, 251–270. 10.1007/s40732-024-00604-1

[CR13] Finn, M., & Barnes-Holmes, D. (2021). In support of reacquainting functional contextualism and interbehaviorism. *Journal of Contextual Behavioral Science,**19*, 1–5. 10.1016/j.jcbs.2020.11.001

[CR14] Finn, M., Barnes-Holmes, D., Hussey, I., & Graddy, J. (2016). Exploring the behavioral dynamics of the implicit relational assessment procedure: The impact of three types of introductory rules. *The Psychological Record,**66*, 309–321. 10.1007/s40732-016-0173-4

[CR15] Finn, M., Barnes-Holmes, D., & McEnteggart, C. (2018). Exploring the single-trial-type-dominance- effect on the IRAP: Developing a differential arbitrarily applicable relational responding effects (DAARRE) model. *The Psychological Record,**68*(1), 11–25. 10.1007/s40732-017-0262-z

[CR16] Finn, M., Barnes-Holmes, D., McEnteggart, C., & Kavanagh, D. (2019). Predicting and influencing the single trial-type dominance effect. *The Psychological Record,**69*(3), 425–435. 10.1007/s40732-019-00347-4

[CR17] Fryling, M., & Hayes, L. J. (2011). The concept of function in the analysis of behavior. *Mexican Journal of Behavior Analysis,**37*, 11–20. 10.5514/rmac.v37.i1.24686

[CR18] Harte, C., Barnes-Holmes, D., de Rose, J. C., Perez, W. F., & de Almeida, J. H. (2023). Grappling with the complexity of behavioral processes in human psychological suffering: Some potential insights from relational frame theory. *Perspectives on Behavior Science,**46*, 237–259. 10.1007/s40614-022-00363-w 37006604 10.1007/s40614-022-00363-w PMC10050485

[CR19] Harte, C., & Barnes-Holmes, D. (2024). Recent developments in RFT encourage interbehavioral field-based views of human language and cognition: A preliminary analysis. *Perspectives on Behavior Science,**47*, 675–690. 10.1007/s40614-024-00407-3 39309238 10.1007/s40614-024-00407-3 PMC11410742

[CR20] Hayes, L. J. (1992a). The psychological present. *The Behavior Analyst,**15*, 139–145. 10.1007/BF03392596 22478124 10.1007/BF03392596 PMC2733478

[CR21] Hayes, L. J. (1992b). Equivalence as process. In S. C. Hayes & L. J. Hayes (Eds.), *Understanding verbal relations* (pp. 97–108). Context Press.

[CR22] Hayes, L. J., Adams, M. A., & Dixon, M. R. (1997). Causal constructs and conceptual confusions. *The Psychological Record,**47*, 97–112. 10.1007/BF03395214

[CR23] Hayes, L. J., & Fryling, M. J. (2018). Psychological events as integrated fields. *The Psychological Record,**68*, 273–277. 10.1007/s40732-018-0274-3

[CR24] Hayes, L. J., & Fryling, M. J. (2019). Functional and descriptive contextualism. *Journal of Contextual Behavioral Science,**14*, 119–126. 10.1016/j.jcbs.2019.09.002

[CR25] Hayes, L. J., & Fryling, M. J. (2023). *Interbehaviorism: A comprehensive guide to the foundations of Kantor’s theory and it’s applications for modern behavior analysis.* Context Press.

[CR26] Hayes, L. J., & Fryling, M. J. (2009). Overcoming the pseudo-problem of private events in the analysis of behavior. *Behavior and Philosophy,**37*, 39–57.

[CR27] Hayes, S. C. (1991). A relational control theory of stimulus equivalence. In L. J. Hayes & P. N. Chase (Eds.), *Dialogues on verbal behavior* (pp. 19–40). Context Press.

[CR28] Hayes, S. C., & Barnes-Holmes, D. (2004). Relational operants: Processes and implications: A response to Palmer’s review of relational frame theory. *Journal of the Experimental Analysis of Behavior,**82*(2), 213–224. 10.1901/jeab.2004.82-213 15540506 10.1901/jeab.2004.82-213 PMC1285006

[CR29] Hayes, S. C., Barnes-Holmes, D., & Roche, B. (2001). *Relational frame theory: A post-Skinnerian account of human language and cognition.* Plenum 10.1016/s0065-2407(02)80063-5 11605362

[CR30] Hayes, S. C., Blackledge., J. T., & Barnes-Holmes, D. (2001). Language and cognition: Constructing an alternative approach within the behavioral tradition. In S. C. Hayes, D. Barnes-Holmes, & B. Roche (Eds.), *Relational frame theory: A post-Skinnerian account of human language and cognition* (pp. 3–20). Plenum. 10.1016/s0065-2407(02)80063-5 11605362

[CR31] Hayes, S. C., & Hayes, L. J. (1989). The verbal action of the listener as a basis for rule-governance. In S. C. Hayes (Ed.), *Rule-governed behavior: Cognition, contingencies, and instructional control* (pp. 153–190). Plenum Press.

[CR32] Hayes, S. C. (2001). A personal prologue. In S. C. Hayes, D. Barnes-Holmes, & B. Roche (Eds.), *Relational frame theory: A post-Skinnerian account of human language and cognition* (pp. vii–ix). Plenum. 10.1016/s0065-2407(02)80063-5 11605362

[CR33] Kantor, J. R. (1933). In defense of stimulus–response psychology. *Psychological Review,**40*, 324–336. 10.1037/h0073851

[CR34] Kantor, J. R. (1926). *Principles of psychology* (Vol. 1–2). Principia Press. (Originally published 1924)

[CR35] Kantor, J. R. (1950). *Psychology and logic (Vol*. Principia Press.

[CR36] Kantor, J. R. (1953). *Logic of modern science*. Principia Press.

[CR37] Kantor, J. R. (1957). Events and constructs in the science of psychology. Philosophy: Banished and recalled. *The Psychological Record,**7*, 55–60. 10.1007/BF03393285

[CR38] Kantor, J. R. (1958). *Interbehavioral psychology*. Principia Press.

[CR39] Kantor, J. R. (1977). *Psychological linguistics*. Principia Press.

[CR40] Kantor, J. R., & Smith, N. W. (1975). *The science of psychology: An interbehavioral survey*. Principia Press.

[CR41] Keuleers, E., Diependaele, K., & Brysbaert, M. (2010). Practice effects in large-scale visual word recognition studies: A lexical decision study on 14,000 Dutch mono-and disyllabic words and nonwords. *Frontiers in Psychology,**1*, 174. 10.3389/fpsyg.2010.00174 21833236 10.3389/fpsyg.2010.00174 PMC3153785

[CR42] Midgley, B., & Morris, E. (2006). *Modern perspectives on J*. Context Press.

[CR43] Muñoz-Blanco, M. I., & Hayes, L. J. (2017). Double substitution of non-linguistic perceptual functions. *European Journal of Behavior Analysis,**18*, 52–70. 10.1080/15021149.2016.1205347

[CR44] Observer. (1977). Comments and queries: Stimulus and stimulation: Problems concerning terms and events. *The Psychological Record, 2*, 503–508. 10.1007/BF03394469

[CR45] Parreiras, G. C., da Costa Chimura, W., Harte, C., Barnes-Holmes, D., & Bortoloti, R. (2025). Exploring the dynamics of relational responding using visual and auditory stimuli: Replicating two prominent IRAP effects. *The Psychological Record,**75*, 505–514. 10.1007/s40732-025-00646-z

[CR46] Pidgeon, A., McEnteggart, C., Harte, C., Barnes-Holmes, D., & Barnes-Holmes, Y. (2021). Four self-related IRAPs: Analyzing and interpreting effects in light of the DAARRE model. *The Psychological Record,**71*(3), 397–409. 10.1007/s40732-020-00428-9

[CR47] Pinto, J. A. R., de Almeida, R. V., & Bortoloti, R. (2020). The stimulus’ orienting function may play an important role in IRAP performance: Supportive evidence from an eye-tracking study of brands. *The Psychological Record,**70*, 257–266. 10.1007/s40732-020-00378-2

[CR48] Power, P. M., Harte, C., Barnes-Holmes, D., & Barnes-Holmes, Y. (2017). Exploring racial bias in a European country with a recent history of immigration of Black Africans. *The Psychological Record,**67*, 365–375. 10.1007/s40732-017-0223-6

[CR49] Rachlin, H. (2011). Baum’s private thoughts. *The Behavior Analyst,**34*(2), 209–212. 10.1007/BF03392251 22532742 10.1007/BF03392251 PMC3211380

[CR50] Schmidt, M., de Rose, J. C., & Bortoloti, R. (2021). Relating, orienting and evoking functions in an IRAP study involving emotional pictographs (emojis) used in electronic messages. *Journal of Contextual Behavioral Science,**21*, 80–87. 10.1016/j.jcbs.2021.06.005

[CR51] Smith, N. W. (2007). Events and constructs. *The Psychological Record,**57*, 169–186. 10.1007/BF03395570

